# Glucagon Promotes Gluconeogenesis through the GCGR/PKA/CREB/PGC-1α Pathway in Hepatocytes of the Japanese Flounder *Paralichthys olivaceus*

**DOI:** 10.3390/cells12071098

**Published:** 2023-04-06

**Authors:** Mengxi Yang, Mingzhu Pan, Dong Huang, Jiahuan Liu, Yanlin Guo, Yue Liu, Wenbing Zhang

**Affiliations:** 1 The Key Laboratory of Aquaculture Nutrition and Feeds (Ministry of Agriculture and Rural Affairs), The Key Laboratory of Mariculture (Ministry of Education), Ocean University of China, Qingdao 266003, China; 2 Hunan Engineering Technology Research Center of Featured Aquatic Resources Utilization, Fisheries College, Hunan Agricultural University, Changsha 410128, China; 3 Laboratory for Marine Fisheries Science and Food Production Processes, Qingdao National Laboratory for Marine Science and Technology, Qingdao 266237, China

**Keywords:** *Paralichthys olivaceus*, glucagon, gluconeogenesis, signaling pathway, glucose

## Abstract

In order to investigate the mechanism of glucagon regulation of gluconeogenesis, primary hepatocytes of the Japanese flounder (*Paralichthys olivaceus*) were incubated with synthesized glucagon, and methods based on inhibitors and gene overexpression were employed. The results indicated that glucagon promoted glucose production and increased the mRNA levels of glucagon receptor (*gcgr*), guanine nucleotide-binding protein Gs α subunit (*gnas*), adenylate cyclase 2 (*adcy2*), protein kinase A (*pka*), cAMP response element-binding protein 1 (*creb1*), peroxisome proliferator-activated receptor-γ coactivator 1α (*pgc-1α*), phosphoenolpyruvate carboxykinase 1 (*pck1*), and glucose-6-phosphatase (*g6pc*) in the hepatocytes. An inhibitor of GCGR decreased the mRNA expression of *gcgr*, *gnas*, *adcy2*, *pka*, *creb1*, *pgc-1α*, *pck1*, *g6pc,* the protein expression of phosphorylated CREB and PGC-1α, and glucose production. The overexpression of *gcgr* caused the opposite results. An inhibitor of PKA decreased the mRNA expression of *pgc-1α*, *pck1*, *g6pc*, the protein expression of phosphorylated-CREB, and glucose production in hepatocytes. A CREB-targeted inhibitor significantly decreased the stimulation by glucagon of the mRNA expression of *creb1*, *pgc-1α*, and gluconeogenic genes, and glucose production decreased accordingly. After incubating the hepatocytes with an inhibitor of PGC-1α, the glucagon-activated mRNA expression of *pck1* and g6pc was significantly down-regulated. Together, these results demonstrate that glucagon promotes gluconeogenesis through the GCGR/PKA/CREB/PGC-1α pathway in the Japanese flounder.

## 1. Introduction

Glucose homeostasis is critical to animals. It requires the coordination between glucagon and insulin. In mammals such as humans, pigs, and rats, glucagon is a 29-amino acid peptide encoded by the proglucagon gene (GCG) and produced by the pancreatic α-cells [1]. The action of glucagon is initiated by binding to the glucagon receptor (GCGR). GCGR couples to GTP-binding G protein and leads to the subsequent activation of adenylate cyclase (ADCY) to produce cyclic adenosine monophosphate (cAMP); the rise in cAMP activates protein kinase A (PKA), which in turn activates cAMP response element-binding (CREB) protein. The stimulation of CREB protein up-regulates the transcription of peroxisome proliferator -activated receptor-γ coactivator-1α (PGC-1α), thereby increasing gluconeogenesis by stimulating the gene expression of phosphoenolpyruvate carboxykinase (PCK) and glucose-6-phosphatase (G6PC) [2,3].

Type 2 diabetes (T2D) has become a worldwide metabolic disease. An uncontrolled increase in blood glucose levels is the hallmark of T2D [4,5]. Patients with T2D exhibit persistent hyperglucagonemia [6]. The abnormal secretion and action of glucagon induce insulin resistance to promote the occurrence of T2D and contribute to hyperglycemia in patients [7,8]. In contrast, inhibition of the glucagon receptor by monoclonal antibodies or antisense oligonucleotides or inhibition of glucagon secretion by glucagon-like peptide 1 resulted in a significantly decreased blood glucose concentration compared to that in diabetic subjects lacking glucagon suppression [9,10,11]. Therefore, antagonizing glucagon action, and hence the blockade of glucagon-induced hepatic glucose production, is a strategy for glycemic control and intervention for T2D [3,12].

Many fish species are considered “natural diabetics”, especially carnivorous fish, in which a carbohydrate-enriched diet or glucose administration causes persistent hyperglycemia [13,14,15]. A previous study showed that the carnivorous Chinese longsnout catfish (*Leiocassis longirostris Günther*) exhibited a lower ability to inhibit gluconeogenesis compared to the herbivorous grass carp (*Ctenopharyngodon idella*) after the oral administration of starch [16]. Moreover, Viegas et al. demonstrated that plasma glucose in European sea bass (*Dicentrarchus labrax* L.) is almost entirely derived from gluconeogenesis [17]. In fish such as rainbow trout (*Oncorhynchus mykiss*) and catfish (*Ictalurus melas*), glucagon has been shown to increase the blood glucose levels as a result of gluconeogenesis [18,19]. Therefore, glucagon-regulated gluconeogenesis may be responsible for glucose intolerance in carnivorous fish.

The Japanese flounder (*Paralichthys olivaceus*) represents a relatively clear example of glucose-intolerant fish, which is characterized by prolonged hyperglycemia after glucose loading or a carbohydrate-rich diet [20]. Previous studies identified sensing components [21], transporters [20,22], and metabolic enzymes [20,23] of glucose in Japanese flounder species. As regards hormone regulation, Deng et al. found that the serum insulin levels did not rise significantly in Japanese flounder fed with increased dietary carbohydrate levels from 8% to 24% [20]. Furthermore, the serum glucagon levels and mRNA expression of gluconeogenic genes were significantly higher in Japanese flounder fed a diet with 20% of dietary carbohydrates than in those fed a diet with 12% of carbohydrates [24]. These results suggest that uninhibited glucagon-regulated gluconeogenesis might contribute to glucose intolerance in Japanese flounder. However, how glucagon regulates gluconeogenesis and by what signaling pathway is far from well-established in fish.

Hence, the present study aimed to shed light on the signaling pathway leading to gluconeogenesis regulated by glucagon in Japanese flounder. It contributes to a better understanding of the molecular mechanisms of glucose intolerance in carnivorous fish.

## 2. Materials and Methods

### 2.1. Ethical Statement

All animal procedures were performed according to the recommendations in the Guide for the Use of Experimental Animals of the Ocean University of China.

### 2.2. Cloning and Tissue Distribution Analysis of GCG

Japanese flounder with an average weight of 52.30 ± 8.18 g were purchased from a commercial hatchery (Qingyuan Marine Biological Technology Co., Ltd., Qingdao, China) and stocked at 22 ± 2 °C in tanks (200 L) in an indoor recirculating water system. Prior to the experiment, the fish were fed a commercial diet (Seglin Biological Engineering Co., Ltd., Qingdao, China) and allowed to acclimate for two weeks. After that, the fish were anesthetized with eugenol (Macklin, Shanghai, China) and killed by a sharp blow to the head. The blood was removed to avoid the influence of blood on the liver, which was used to clone the gene for GCG. Liver samples were collected and immediately frozen in liquid nitrogen and then transferred to −80 °C for gene cloning.

Total RNA was extracted from the liver using Trizol RNAiso Plus (Takara Biotech, Dalian, China) according to the manufacturer’s protocol. Then, complementary DNA (cDNA) was synthesized from 1 mg of total RNA using the PrimeScript^®^ RT reagent kit with gDNA Eraser (Takara, Kyoto, Japan). Degenerated primers (Table 1) were designed on the conserved regions of GCG from different species. First-strand cDNA was synthesized by the SMART RACE cDNA amplification kit (Takara, Kyoto, Japan) and used as the template for the 3′ or 5′ rapid amplification of cDNA ends (RACE) PCR. Subsequently, inner and outer primers (Table 1) were further designed for RACE PCR using Primer Premier 5. All PCR products were separated on a 1.2% agarose gel, and the target bands were purified and then ligated into the pEASY-T1 vector (TransGen, Beijing, China). The PCR products were sequenced by Sangon Biotech (Shanghai, China) [25].

The brain, liver, stomach, kidney, intestine, spleen, heart, and muscle of Japanese flounder were used to analyze the tissue distribution of GCG.

### 2.3. Sequence Analysis and 3D Structure of GCG

All nucleotide sequences were blasted using the BlastX algorithm at the National Center for Biotechnology Information to determine gene identity. The Open Reading Frame of GCG was identified by ORF finder, and the cDNA sequence was translated. The protein domain was predicted by SMART. The GCG gene sequences of different species, including fish, were selected in the GenBank, and ClustalW software version 2.1 (University College Dublin, Dublin, Ireland) was used for multiple sequence comparison analysis. A phylogenetic tree was constructed based on multiple sequence alignments using the neighbor-joining method with the MEGA 5.0 program. The protein structure model was predicted using the SWILL-MODLE workspace.

### 2.4. Synthesis of Glucagon

According to the deduced amino acid sequence, the Japanese founder glucagon peptide with 29 amino acids was synthesized using the solid-phase synthetic peptide method by Sangon Biotech (Shanghai, China). The molecular weight of this peptide was detected by liquid chromatography–mass spectrometry, and its purity was detected by high-performance liquid chromatography.

### 2.5. Treatments with Glucagon and Inhibitors in Hepatocytes

Hepatocytes were isolated from the liver of Japanese flounder and cultured as described previously with some modifications [26]. Japanese flounder with an average weight of 38.52 ± 7.23 g were acclimated and kept in seawater containing 100 U mL^−1^ of penicillin (Gibco, Grand Island, NY, USA) and 100 μg mL^−1^ of streptomycin (Gibco, Grand Island, NY, USA) for 24 h. At the start of the experiment, the fish were anesthetized with eugenol (1:10,000 dilution with seawater) and then bloodletting for 5 min. After the fish surface was dried with a towel and disinfected with 75% (*v*/*v*) alcohol, the livers of five fish were excised and washed three times with cold phosphate-buffered saline (PBS) (Solarbio, Beijing, China) containing 100 IU mL^−1^ of penicillin–streptomycin (Gibco, Grand Island, NY, USA). The liver fragments were diced to 1 mm^3^ in Dulbecco’s Modified Eagle Medium/Nutrient Mixture F-12 (DMEM/F12) (Gibco, Grand Island, NY, USA) and then digested with 0.25% trypsin–EDTA (Gibco, Grand Island, NY, USA) for 15 min. During the digestion, the supernatant was collected every 5 min by a Pasteur pipette (Corning Inc., Corning, NY, USA) in 2 volumes of DMEM/F12 medium containing 15% fetal bovine serum (FBS; Gibco, Grand Island, NY, USA). Then, the isolated hepatocytes were purified through a sterile 40 μm mesh and harvested by centrifugation at 1000× *g* for 5 min at 4 °C. Red blood cell lysis buffer (Gibco, Grand Island, NY, USA) was used to eliminate the red blood cells. After washing twice with PBS for debris removal, the purified hepatocytes were re-suspended in the DMEM/F12 medium containing 15% FBS, 1% L-glutamine (Gibco, Grand Island, NY, USA), and 1% antibiotics. The hepatocytes were seeded in 6-well plates (Corning Inc., Corning, NY, USA) at a density of 1.0 × 10^6^ cells/well and placed at 23 °C in a normal-atmosphere incubator overnight. The culture medium was refreshed every 2 days until the cells became 80–90% confluent. After the removal of the old culture medium, the test substances prepared in DMEM/F12 medium (the glucose concentration was 17.5 mM) were gently overlaid onto the hepatocytes for further studies.

For glucagon incubation at different time intervals, the hepatocytes were incubated with 1 μM synthetic glucagon (resuspended in DMSO). Sampling occurred at 0 (before the treatment), 3, 9, 12, 18, 24, 30, 36, and 48 h. For glucagon incubation at different concentrations, six glucagon concentrations (0.1 nM, 1 nM, 10 nM, 100 nM, 1 μM, and 10 μM) were used, incubating the hepatocytes for 24 h. The groups with the same respective amount of DMSO were regarded as the control.

For the treatments with the inhibitors, the hepatocytes were incubated with adomeglivant, targeting GCGR (50 μM, HY-19904, MedChemExpress, Monmouth Junction, NJ, USA), H89, targeting PKA (20 μM, HY-15979, MedChemExpress, Monmouth Junction, NJ, USA), 666-15, targeting CREB (40 μM, HY-101120, MedChemExpress, Monmouth Junction, NJ, USA), and SR-18292, targeting PGC-1α (20 μM, HY-101491, MedChemExpress, Monmouth Junction, NJ, USA) for 24 h. Four independent treatments (using one inhibitor per treatment) were performed, and each treatment included four groups, i.e., DMSO, glucagon (1 μM), inhibitor, and glucagon plus inhibitor. For the glucagon-plus-inhibitor group, glucagon was added after the hepatocytes were treated with the inhibitor for 2 h. The group with an equal volume of DMSO was regarded as the control. Before these treatments, the concentrations of the inhibitors were optimized to obtain the highest inhibition efficiency and the low cytotoxicity.

### 2.6. Overexpression of gcgr

The *gcgr* gene coding sequence (CDS) was amplified using specific primers (Table 1). The template *gcgr* was PCR-amplified using primers with homology arms (Table 1) to the BamHI region in pcDNA3.1-EGFP. The resulting amplicons were purified by gel electrophoresis and extracted using the SanPrep Column DNA Gel Extraction Kit (Sangon Biotech, Shanghai, China). The pcDNA3.1-EGFP plasmid (Biofeng, Shanghai, China) was digested with the enzyme BamHI. The purified resulting amplicons were then ligated into the pcDNA3.1-EGFP plasmid using T4 DNA ligase (TransGen, Beijing, China). The plasmid constructed was called pcDNA3.1-GCGR-EGFP. The construct was transformed into the *Escherichia coli* strain DH5α and then confirmed by DNA sequencing. The bacteria successfully transformed with the target gene were expanded by shaking the flask culture overnight, and adequate plasmid for transfection was collected using the EasyPure HiPure Plasmid MaxiPrep Kit (TransGen, Beijing, China).

Primary hepatocytes were seeded in 6-well plates at a density of 1.0 × 10^6^ cells/well. When the hepatocytes were at 80–90% confluency, the cells were transfected with pcDNA3.1-GCGR-EGFP using 3.75 μL/well of Lipofectamine 3000 (Invitrogen, Waltham, MA, USA) according to the manufacturer’s instructions. For each well, 5 μg of plasmid was used. The pcDNA3.1-EGFP plasmid served as a negative control for the experiment. No plasmid but an equal volume of PBS was added in the control group. After 48 h, the transfection efficiency was determined using a fluorescence microscope (Echo Laboratories, USA) and qPCR. The number of EGFP-positive cells was calculated using Image-Pro Plus 6.0 software (Media Cybernetics, USA). The transfection experiments were performed in triplicate.

### 2.7. Measurements of Metabolites

The medium of the hepatocytes was collected. The glucose concentration was determined using a glucose assay kit (F006-1-1) by the glucose oxidase method [27]. The concentration of lactate was determined using a lactate assay kit (A019-2) by the lactate dehydrogenase method [28]. The pyruvate concentration was measured with a pyruvate assay kit (A081-1-1) at an absorbance of 505 nm and calculated with reference to a pyruvate standard sample [29]. All these kits were purchased from Jiancheng Bioengineering Institute, Nanjing, China.

Glucose production was determined as previously described [30]. After incubation of the hepatocytes with appropriate concentrations of the test substances, the culture medium was replaced with 1 mL of glucose-free DMEM without phenol red (Gibco, Grand Island, NY, USA), supplemented with 0.5% BSA, 20 mM sodium lactate, and 2 mM sodium pyruvate. After 6 h of incubation, the medium was collected, and glucose concentration was measured using a glucose oxidation kit (Jiancheng Bioengineering Institute, Nanjing, China).

### 2.8. Real-Time Quantitative RT-PCR Analysis

The gene expression analysis was carried out using the SYBR Green Real-time PCR Master Mix (Q711-02, Vazyme Biotech, Nanjing, China) in a quantitative thermal cycler (Quant Studio 5, Applied Biosystems, Waltham, MA, USA) with the primers shown in Table 1. The mRNA levels were normalized to those of *β-actin* as a housekeeping gene. The data were analyzed by the ΔΔCt method. Before the detection, the amplification efficiency of the primers for each gene was evaluated by serial dilutions to ensure that it was close to 100%.

### 2.9. Western Blot Analysis

The procedures of Western blot analysis were performed as previously described [31]. The following antibodies were used: antibodies against phospho-CREB (Ser^133^) (dilution 1:1000, cat. No. ab32096, Abcam, Cambridge, UK), total CREB (dilution 1:1000, cat. No. ab32515, Abcam, Cambridge, UK), PGC-1α (dilution 1:800, cat. No. AF7736, Beyotime Biotechnology, Shanghai, China), GCGR (dilution 1:800, cat. No. ab75240, Abcam, Cambridge, UK), PCK1 (dilution 1:1000, cat. No. AF7689, Beyotime Biotechnology, Shanghai, China), phospho-PKA Substrate (dilution 1:1000, cat. No. 9624S, Cell Signaling Technologies, Boston, MA, USA), and β-actin (dilution 1:5000, cat. No. bs-0061R, Bioss Antibodies, Beijing, China). All the antibodies were raised against orthologs that share amino acid similarities to the proteins in the Japanese flounder. The detection of Western blot bands of the correct sizes by these antibodies was confirmed. After antibody incubation, HRP-conjugated goat anti-rabbit (dilution 1:5000, cat. No. A0208, Beyotime Biotechnology, Shanghai, China) or goat anti-mouse IgG (dilution 1:5000, cat. No. A0216, Beyotime Biotechnology, Shanghai, China) was added, and the ECL reagent (Beyotime Biotechnology, Shanghai, China) was used as an HRP substrate for signal development. The Western blot bands were quantified using NIH Image 1.63 software.

### 2.10. Statistics Analysis

All data are presented as means ± standard error and were analyzed using the software SPSS 25.0. Two-tailed *t*-test, one-way ANOVA with Tukey post-hoc test, or two-way ANOVA with Tukey post-hoc test was used to compare the means between groups, as indicated in the Figure legends. *p* < 0.05 was considered significant.

## 3. Results

### 3.1. Gene Cloning, Functional Analysis, and Tissue Distribution of GCG and Synthesis of Glucagon

**Figure 1:**
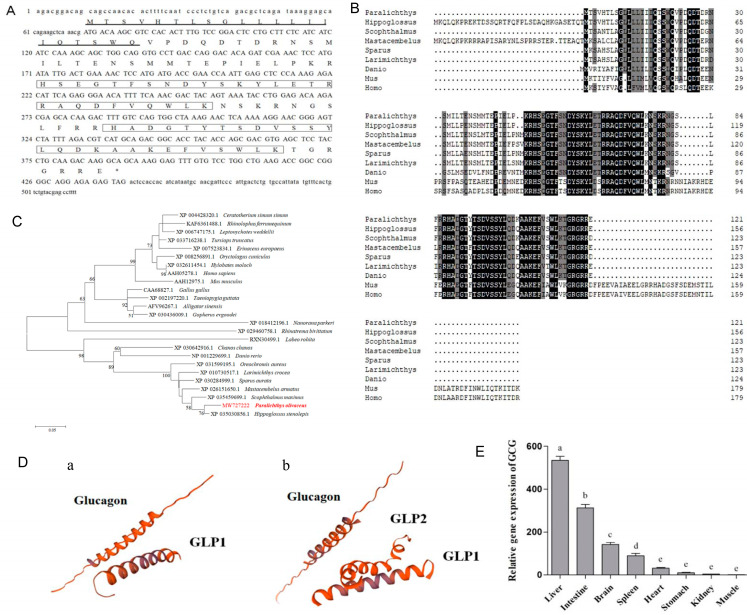
Gene cloning, functional analysis, and tissue distribution of GCG. (**A**), Nucleotide sequence, deduced amino acid sequence, and predicted domains of the GCG gene. The signal peptide is marked with a black underline; the GLUCA domain is marked with a black box; The asterisk (*) indicatesthe stop codon. (**B**), Comparison of the amino acid sequences of GCG. Comparison of the deduced amino acid (aa) sequence of GCG from Japanese flounder with those from other fish, mouse, and human. The aa sequences were aligned using ClustalW, and similarity shading was based on a 75% identity threshold. Identical residues are shaded dark, and similar residues are shaded light. Accession numbers: *Hippoglossus stenolepis* (XP_035030856.1); *Scophthalmus maximus* (XP_035459699.1); *Mastacembelus armatus* (XP_026151650.1); *Sparus aurata* (XP_030284999.1); *Larimichthys crocea* (XP_010730517.1); *Danio rerio* (NP_001229699.1); *Mus musculus* (AAH12975.1); Homo sapiens (AAH05278.1). (**C**), NJ phylogenetic tree based on amino acid sequences. The phylogenetic tree is based on these sequences: *Ceratotherium simum simum* (XP_004428320.1); *Rhinolophus ferrumequinum* (KAF6361488.1); *Leptonychotes weddellii* (XP_006747175.1); *Tursiops truncates* (XP_033716238.1); *Erinaceus europaeus* (XP_007523834.1); *Oryctolagus cuniculus* (XP_008256891.1); *Hylobates moloch* (XP_032611454.1); Homo sapiens (AAH05278.1); *Mus musculus* (AAH12975.1); *Gallus gallus* (CAA68827.1); *Taeniopygia guttata* (XP_002197220.1); *Alligator sinensis* (AFV96267.1); *Gopherus evgoodei* (XP_030436009.1); *Nanorana parkeri* (XP_018412196.1); *Rhinatrema bivittatum* (XP_029460758.1); *Labeo rohita* (RXN30499.1); *Chanos chanos* (XP_030642916.1); *Danio rerio* (NP_001229699.1); *Oreochromis aureus* (XP_031599195.1); *Larimichthys crocea* (XP_010730517.1); *Sparus aurata* (XP_030284999.1); *Mastacembelus armatus* (XP_026151650.1); *Scophthalmus maximus* (XP_035459699.1); *Paralichthys olivaceus* (MW727222); *Hippoglossus stenolepis* (XP_035030856.1); The red words represent Japanese flounder. (**D**), Schematic diagram of the three-dimensional structure of the GCG protein from Japanese flounder and human. (**a**) GCG in Japanese flounder; (**b**) GCG in human. (**E**), Tissues distribution of GCG in Japanese flounder. The values represent the means ± S.E., *n* = 3, one-way ANOVA with Tukey post-hoc test. Values with different letters mean statistical differences (*p <* 0.05).

The nucleotide sequence analysis of the putative GCG cDNA revealed an open reading frame of 366 bp, encoding 121 amino acids (aa), with a signal peptide (aa 1–21) and two GLUCA domains (aa 50–76 and aa 88–114) (Figure 1A). The predicted molecular weight is 14 kDa, and the theoretical isoelectric point is 9.07. Comparison of this amino acid sequence with the corresponding sequences of the known vertebrate GCG indicated that it shares the highest sequence identity with the GCG of Pacific halibut (*Hippoglossus stenolepis*) and turbot (*Scophthalmus maximus*) (96.69% and 91.87%, respectively) (Figure 1B). A phylogenetic analysis showed the GCG of the Japanese flounder has the strongest relationship with fish, followed by amphibians, reptiles, birds, and mammals (Figure 1C). By predicting the three-dimensional structure of the GCG protein in the Japanese flounder, the two protein structures of glucagon (aa 50–78) and glucagon-like peptide 1 (aa 88–118) were simulated at different positions (Figure 1D). A tissue expression analysis illustrated that the GCG gene is primarily expressed in the liver, followed by the intestine and brain (Figure 1E).

Glucagon of the Japanese flounder was then chemically synthesized according to the amino acid sequence HSEGTFSNDYSKYLETRRAQDFVQWLKNS. For synthetic glucagon, the molecular weight was 3507.00, and the purity was more than 98%.

### 3.2. Glucagon Induces Gene Expression in Hepatocytes and Increases the Glucose Concentration in the Medium

**Figure 2:**
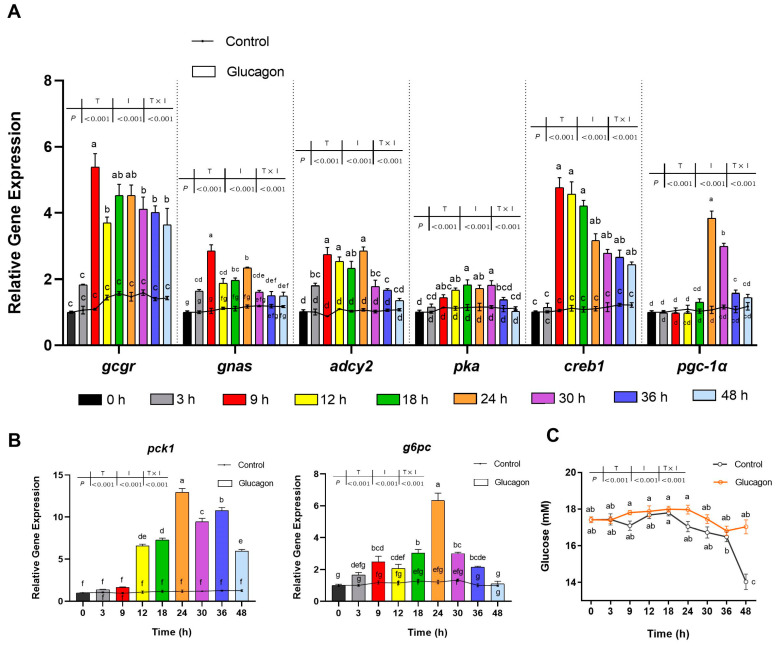
Time-dependent effects of glucagon treatment on gene expression in hepatocytes and glucose concentration in the medium. (**A**), qPCR analysis of the mRNA levels of glucagon pathway-related genes (*gcgr*; *gnas*; *adcy2*; *pka*; *creb1*; *pgc-1α*) in hepatocytes at different times in response to glucagon treatment (1 μM) or DMSO. (**B**), qPCR analysis of the mRNA levels of gluconeogenic genes (*pck1* and *g6pc*) in hepatocytes at different times in response to glucagon treatment (1 μM) or DMSO. (**C**), Glucose concentration in the medium at different times in response to glucagon treatment (1 μM) or DMSO. All data are expressed as mean ± SE, *n* = 3, two-way ANOVA with Tukey post-hoc test. Values with different letters mean statistical differences (*p* < 0.05). T: time points; I: reagents for incubation; T × I: interaction between time and reagents.

As shown in Figure 2A, the mRNA levels of *gcgr*, guanine nucleotide-binding protein Gs subunit alpha (*gnas*), *adcy2*, *pka*, *creb1*, and *pgc-1α* were stimulated by glucagon incubation in a time-dependent manner. The mRNA levels of *gnas* and *adcy2* were up-regulated significantly at 3 h compared to 0 h (*p* < 0.05) and reached their maximum values at 9 h and 24 h, respectively. Compared with 0 h, the *gcgr*, *pka*, and *creb1* mRNA levels were significantly up-regulated at 9 h (*p* < 0.05). The maximal effect of glucagon on *pgc-1α* mRNA levels was observed at 24 h after treatment. As shown in Figure 2B, the mRNA levels of *pck1* and *g6pc* in the glucagon group were higher than those in the control group after treatment. Both genes were up-regulated as the time increased, and the maximal stimulatory responses for them following glucagon incubation were observed at 24 h. Then, the mRNA levels of *pck1* and *g6pc* declined. The glucose concentration in the medium of the glucagon group was higher than that in the medium of the control group after incubation. It was significantly higher than that in the medium of the control group at 48 h (*p* < 0.05) (Figure 2C).

**Figure 3:**
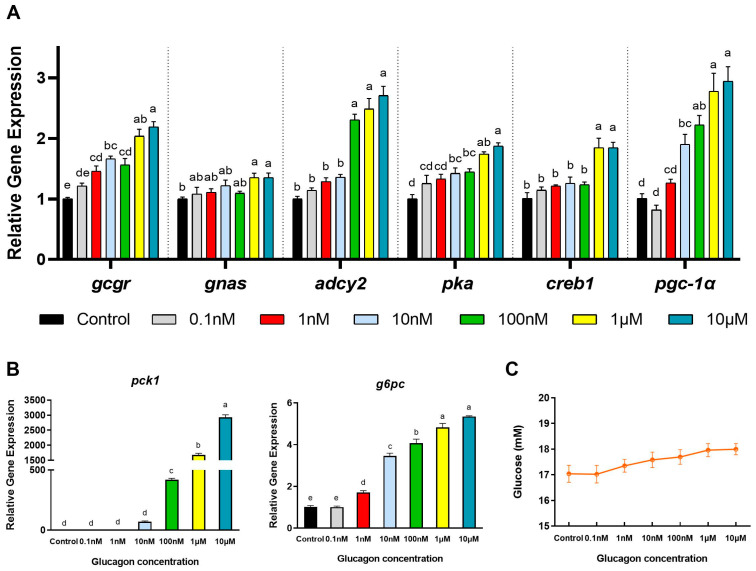
Dose-dependent effects of glucagon treatment on the mRNA levels of genes in hepatocytes and glucose concentration in the medium. (**A**), qPCR analysis of the mRNA levels of glucagon pathway-related genes (*gcgr*; *gnas*; *adcy2*; *pka*; *creb1*; *pgc-1α*) in hepatocytes in response to glucagon treatment at different doses or DMSO (24 h). (**B**), qPCR analysis of the mRNA levels of gluconeogenic genes (*pck1* and *g6pc*) in hepatocytes in response to glucagon treatment at different doses or DMSO (24 h). (**C**), Glucose concentration in the medium in response to glucagon treatment at different doses or DMSO (24 h). All data are expressed as mean ± SE, *n* = 3, one-way ANOVA with Tukey post-hoc test. Values with different letters mean statistical differences (*p* < 0.05).

Figure 3A shows that increasing concentrations of glucagon could elevate the mRNA levels of glucagon pathway-related genes in a dose-dependent manner. Compared with the control group, the *gcgr* mRNA levels were significantly up-regulated when the glucagon concentration was 1 nM (*p* < 0.05). Treatment with 10 nM glucagon significantly increased the *pka* and *pgc-1α* mRNA levels (*p* < 0.05). The mRNA levels of *adcy2* were up-regulated markedly in the presence of 100 nM glucagon (*p* < 0.05). The mRNA levels of *gnas* and *creb1* were up-regulated significantly by 1 μM glucagon (*p* < 0.05). The maximal response of all measured genes to glucagon was observed using a dose of 10 μM glucagon. As shown in Figure 3B, compared with the control group, the *pck1* mRNA level was up-regulated markedly by 100 nM glucagon (*p* < 0.05) and reached the maximum value at 10 μM. For *g6pc*, its mRNA level was significantly up-regulated by 1 nM glucagon (*p* < 0.05) and peaked at 10 μM glucagon. The glucose concentration in the medium showed an upward trend with the increase of glucagon concentration, but no significant difference was observed (*p* > 0.05) (Figure 3C).

### 3.3. Adomeglivant Inhibits the Glucagon Pathway in Hepatocytes by Targeting GCGR

**Figure 4:**
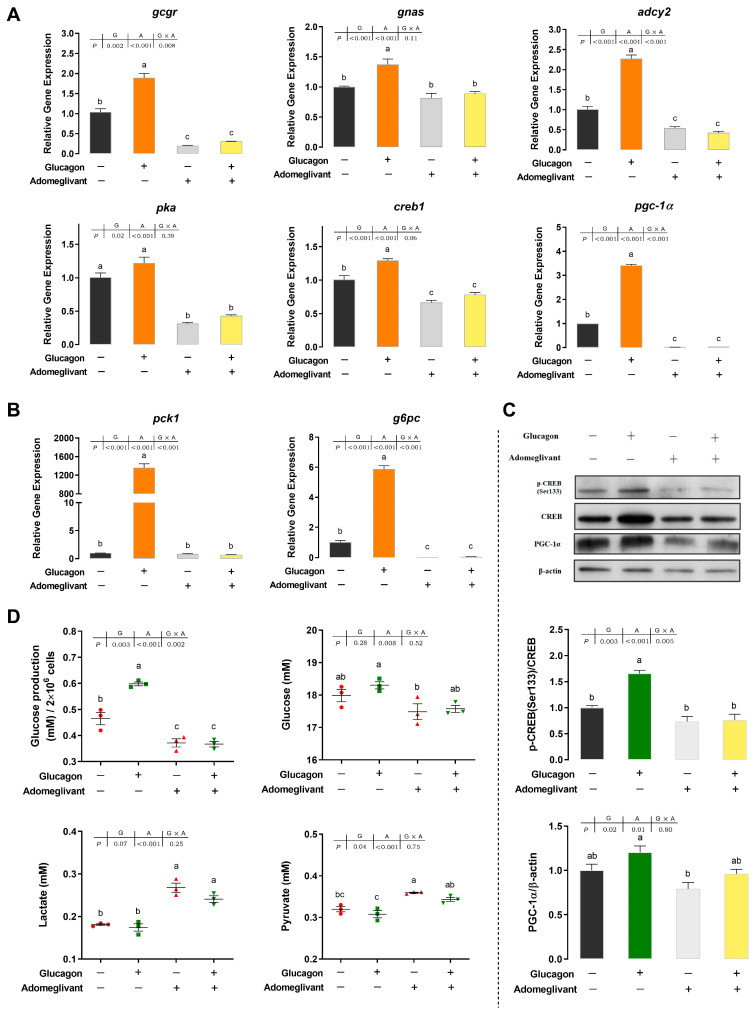
Adomeglivant inhibits the glucagon pathway in hepatocytes by targeting GCGR. (**A**), qPCR analysis of the mRNA levels of glucagon pathway-related genes (*gcgr*; *gnas*; *adcy2*; *pka*; *creb1*; *pgc-1α*) following glucagon stimulation and treatment with adomeglivant (50 μM) in hepatocytes. (**B**), qPCR analysis of the mRNA levels of gluconeogenic genes (*pck1* and *g6pc*) following glucagon stimulation and treatment with adomeglivant (50 μM) in hepatocytes. (**C**), Western blot analysis of p-CREB (Ser^133^) and PGC-1α following glucagon stimulation and treatment with adomeglivant (50 μM) in hepatocytes. (**D**), Glucose production in hepatocytes, glucose, lactate, and pyruvate concentrations in the medium following glucagon stimulation and treatment with adomeglivant (50 μM). All data are expressed as mean ± SE, *n* = 3, two-way ANOVA with Tukey post-hoc test. Values with different letters mean statistical differences (*p* < 0.05). G: glucagon treatments; A: adomeglivant treatments; G × A: interaction between glucagon and adomeglivant treatments.

As shown in Figure 4A, compared with the control group, adomeglivant significantly reduced the *gcgr* mRNA levels (*p* < 0.05) and abolished the increase of *gcgr* mRNA levels induced by glucagon (*p* < 0.05). The mRNA levels of *adcy2*, *pka*, *creb1*, and *pgc-1α* showed similar trends as those of *gcgr*. The *gnas* mRNA levels were significantly lower in the group treated with both glucagon and adomeglivant than in the group only incubated with glucagon (*p* < 0.05). No significant difference was observed between the control group and the adomeglivant group (*p* > 0.05). As shown in Figure 4B, the mRNA levels of *pck1* showed similar trends as those of *gnas*. Adomeglivant significantly decreased the *g6pc* mRNA levels compared with the control group (*p* < 0.05). Following pre-incubation with adomeglivant, the stimulatory effects of glucagon on the *g6pc* mRNA levels were abolished (*p* < 0.05). As shown in Figure 4C, glucagon was effective at triggering the phosphorylation of CREB (*p* < 0.05). The glucagon-induced phosphorylated CREB protein expression could be blocked by pre-treatment with the adomeglivant (*p* < 0.05). Compared with the control group, PGC-1α protein expression showed an upward trend following glucagon incubation and a downward trend following adomeglivant treatment (*p* > 0.05). It also tended to decrease following glucagon-plus-adomeglivant treatment compared with glucagon treatment (*p* > 0.05). As shown in Figure 4D, compared with the control group, glucagon significantly increased the glucose production in hepatocytes (*p* < 0.05), but the glucose concentration in the medium was not significantly affected (*p* > 0.05). Incubation with adomeglivant significantly decreased the glucose production in hepatocytes (*p* < 0.05) and did not significantly increase the glucose concentration in the medium (*p* > 0.05). As for the gluconeogenic substrates in the medium, the lactate and pyruvate concentrations significantly increased folllowing adomeglivant incubation (*p* < 0.05).

### 3.4. Overexpression of Gcgr Activates the Glucagon Pathway in Hepatocytes

**Figure 5:**
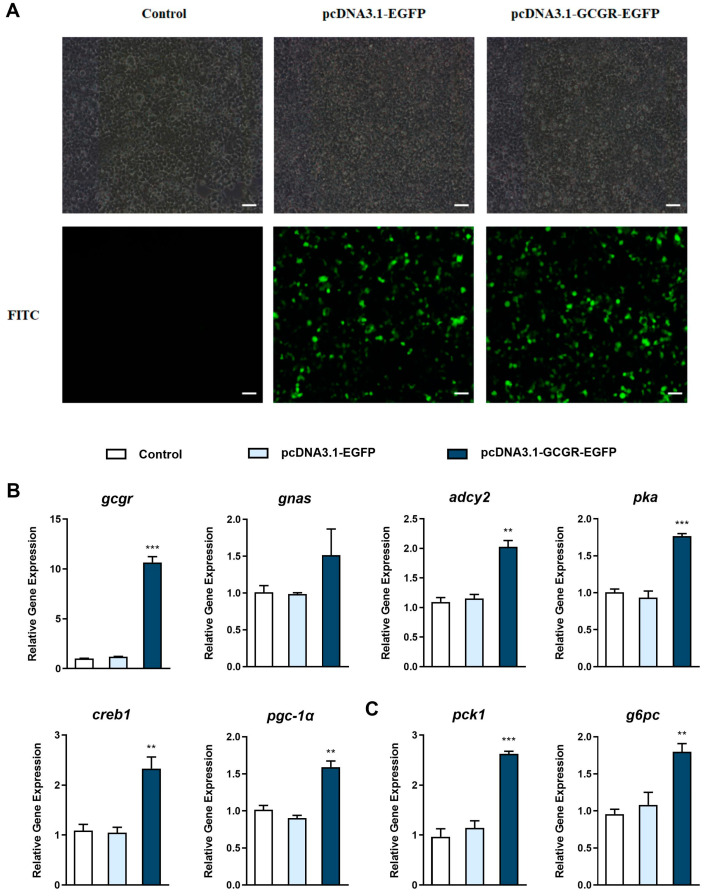

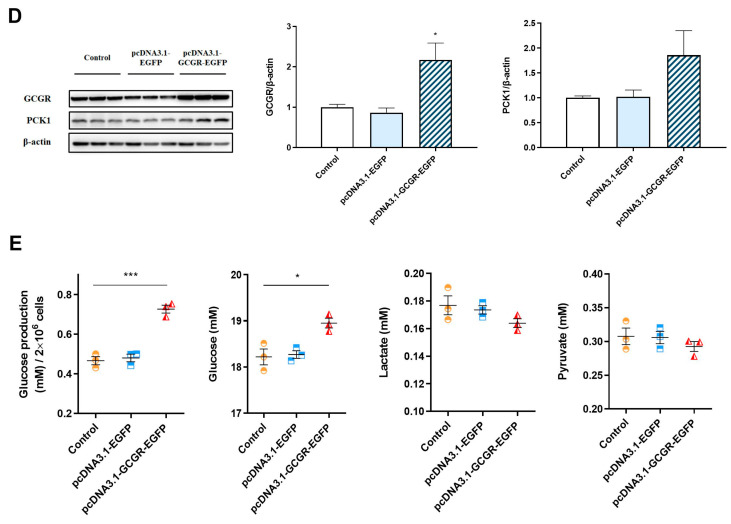
Overexpression of *gcgr* activates the glucagon pathway in hepatocytes. (**A**), Images of hepatocytes 48 h after transfection; bar = 100 µm. The cells were treated with PBS and transfected with the pcDNA3.1-EGFP plasmid and the pcDNA3.1-GCGR-EGFP plasmid. (**B**), qPCR analysis of the mRNA levels of glucagon pathway -elated genes (*gcgr*; *gnas*; *adcy2*; *pka*; *creb1*; *pgc-1α*) in hepatocytes treated with PBS and transfected with the pcDNA3.1-EGFP plasmid and the pcDNA3.1-GCGR-EGFP plasmid. (**C**), qPCR analysis of the mRNA levels of gluconeogenic genes (*pck1* and *g6pc*) in hepatocytes treated with PBS and transfected with the pcDNA3.1-EGFP plasmid and the pcDNA3.1-GCGR-EGFP plasmid. (**D**), Western blot analysis of GCGR and PCK1 in hepatocytes treated with PBS and transfected with the pcDNA3.1-EGFP plasmid and the pcDNA3.1-GCGR-EGFP plasmid. (**E**), Glucose production in hepatocytes and glucose, lactate, and pyruvate concentrations in the medium following treatment with PBS and transfection with the pcDNA3.1-EGFP plasmid and the pcDNA3.1-GCGR-EGFP plasmid. All data are expressed as mean ± SE, *n* = 3, two-tailed *t*-test. * *p* < 0.05, ** *p* < 0.01, *** *p* < 0.001 versus PBS control.

Images of the hepatocytes 48 h after transfection are shown in Figure 5A. The percentage of EGFP-positive cells was 31.01 ± 1.52% in the pcDNA3.1-EGFP group and 30.51 ± 1.07% in the pcDNA3.1-MSTN-1-EGFP group. Compared with the control group, transfection with the pcDNA3.1-EGFP plasmid did not influence the mRNA expression of *gcgr* (*p* > 0.05), while *gcgr* overexpression via pcDNA3.1-GCGR-EGFP transfection significantly increased the mRNA levels of *gcgr* by about 10 times (*p* < 0.001) (Figure 5B). Compared with the control group, 48 h after transfecting with pcDNA3.1-GCGR-EGFP, the mRNA levels of *adcy2*, *pka*, *creb1*, *pgc-1α*, *pck1*, and *g6pc* (Figure 5C) were up-regulated significantly (*p* < 0.01), while the *gnas* mRNA levels were up-regulated but not significantly (*p* > 0.05). The protein expression of GCGR and PCK1 was also up-regulated in the pcDNA3.1-GCGR-EGFP group compared with the control group (Figure 5D). Compared with the control group, glucose production in the hepatocytes and glucose concentration in the medium increased significantly in the pcDNA3.1-GCGR-EGFP group (Figure 5E) (*p* < 0.05), but the lactate and pyruvate concentrations in the medium tended to decrease, though not significantly (*p* > 0.05).

### 3.5. H89 Inhibits the Glucagon Pathway in Hepatocytes by Targeting PKA

**Figure 6:**
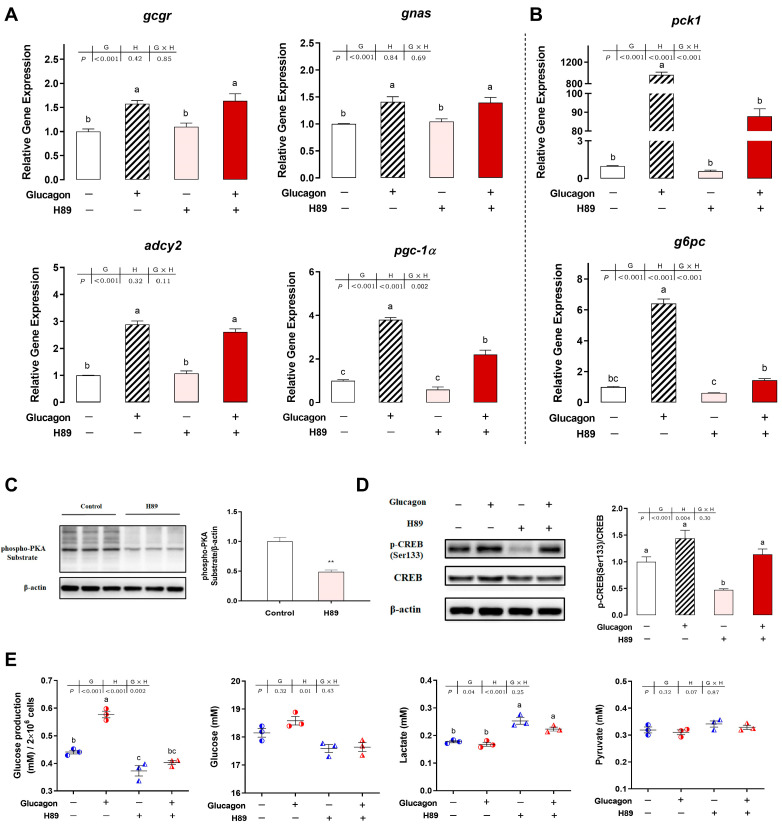
H89 inhibits the glucagon pathway in hepatocytes by targeting PKA. (**A**), qPCR analysis of the mRNA levels of glucagon pathway-related genes (*gcgr*; *gnas*; *adcy2*; *pgc-1α*) following glucagon stimulation and treatment with H89 (20 μM) in hepatocytes. (**B**), qPCR analysis of the mRNA levels of gluconeogenic genes (*pck1* and *g6pc*) following glucagon stimulation and treatment with H89 (20 μM) in hepatocytes. (**C**), Western blot analysis of a phospho-PKA substrate treated with H89 (20 μM) in hepatocytes. (**D**), Western blot analysis of p-CREB (Ser^133^) following glucagon stimulation and treatment with H89 (20 μM) in hepatocytes. (**E**), Glucose production in hepatocytes and glucose, lactate, and pyruvate concentrations in the medium following glucagon stimulation and treatment with H89 (20 μM). All data are expressed as mean ± SE, *n* = 3. For (**A**,**B**,**D**,**E**), *p* values were determined by two-way ANOVA with Tukey post-hoc test. Values with different letters mean statistical differences (*p* < 0.05). G: glucagon treatments; H: H89 treatments; G × H: interaction between glucagon and H89 treatments. For C, *p* values were determined by two-tailed *t*-test. ** *p* < 0.01.

As shown in Figure 6A,B, compared with the control group, glucagon incubation significantly increased the mRNA levels of the measured genes. H89 incubation did not influence the mRNA levels of *gcgr*, *gnas*, *adcy2*, *pgc-1α*, *pck1*, and *g6pc* compared with the control group (*p* > 0.05). Compared with the glucagon treatment alone, pre-treatment with H89 had no influence on the mRNA levels of *gcgr*, *gnas*, and *adcy2* (*p* > 0.05) but significantly decreased the mRNA levels of *pgc-1α*, *pck1*, and *g6pc* in the hepatocytes (*p* < 0.05). Compared with the control group, the phosphorylation levels of a PKA substrate (Figure 6C) and CREB at Ser133 (Figure 6D) were down-regulated significantly by H89 incubation (*p* < 0.01). Compared with the glucagon group, pre-treatment with H89 decreased the levels of phosphorylated CREB (Ser133) (*p* > 0.05). As shown in Figure 6E, compared with the control group, H89 significantly decreased glucose production in the hepatocytes but increased the lactate concentration in the medium (*p* < 0.05). No significant differences were observed in glucose and pyruvate concentrations with the different treatments (*p* > 0.05).

### 3.6. The Molecule 666-15 Inhibits the Glucagon Pathway in Hepatocytes by Targeting CREB

**Figure 7:**
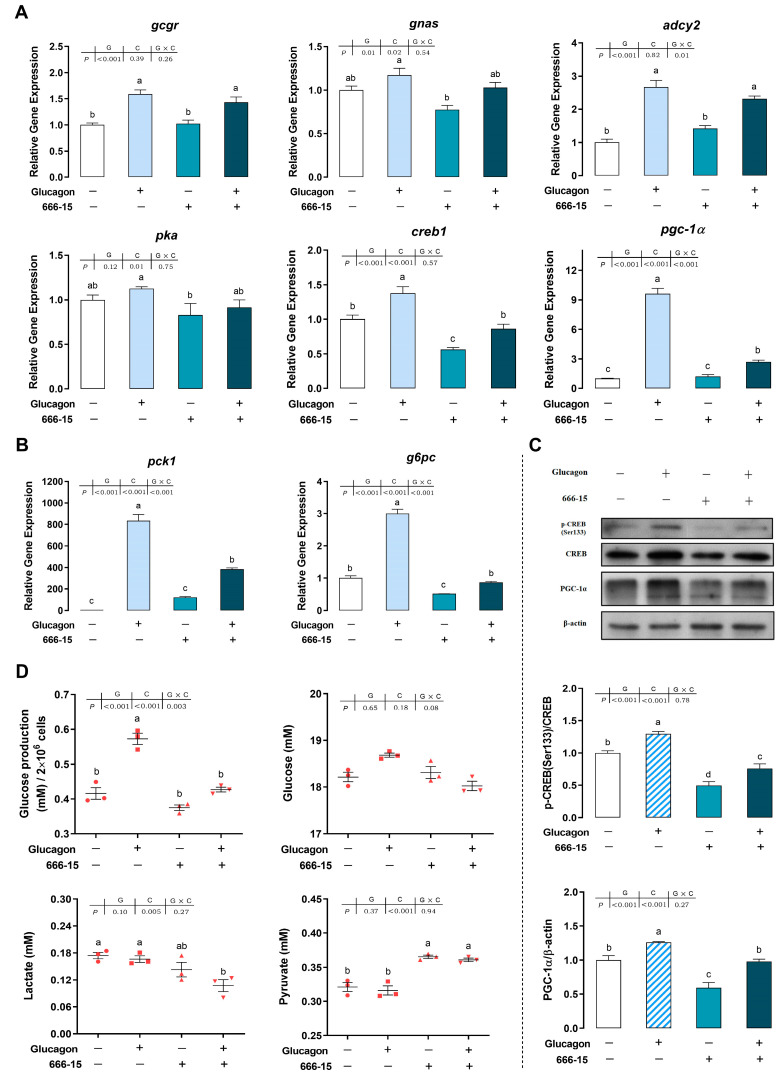
The compound 666-15 inhibits the glucagon pathway in hepatocytes by targeting CREB. (**A**), qPCR analysis of the mRNA levels of glucagon pathway-related genes (*gcgr*; *gnas*; *adcy2*; *pka*; *creb1*; *pgc-1α*) following glucagon stimulation and treatment with 666-15 (40 μM) in hepatocytes. (**B**), qPCR analysis of the mRNA levels of gluconeogenic genes (*pck1* and *g6pc*) following glucagon stimulation and treatment with 666-15 (40 μM) in hepatocytes. (**C**), Western blot analysis of p-CREB (Ser^133^) and PGC-1α following glucagon stimulation and treatment with 666-15 (40 μM) in hepatocytes. (**D**), Glucose production in hepatocytes and glucose, lactate, and pyruvate concentrations in the medium following glucagon stimulation and treatment with 666-15 (40 μM). All data are expressed as mean ± SE, *n* = 3, two-way ANOVA with Tukey post-hoc test. Values with different letters mean statistical differences (*p* < 0.05). G: glucagon treatments; C: 666-15 treatments; G × C: interaction between glucagon and 666-15 treatments.

As shown in Figure 7A,B, compared with the control group, glucagon incubation increased the mRNA levels of the measured genes. Incubation of the hepatocytes with an inhibitor of CREB (666-15) significantly decreased the *creb1* mRNA levels compared with the control group (*p* < 0.05). Following pre-treatment with 666-15, the stimulatory effects of glucagon on *creb1* mRNA levels were abolished (*p* < 0.05). The mRNA levels of *g6pc* showed a similar trend as the *creb1* mRNA levels. Compared with the control group, 666-15 incubation had no effect on the mRNA levels of *gcgr*, *gnas*, *adcy2*, *pka*, *pgc-1α*, and *pck1* (*p* > 0.05). Furthermore, the mRNA levels of *gcgr*, *gnas*, *adcy2*, and *pka* did not change significantly in the glucagon-plus-666-15 group compared with those in the glucagon group (*p* > 0.05). Regarding *pgc-1α* and *pck1*, 666-15 abolished the stimulatory effects of glucagon on their mRNA levels (*p* < 0.05). Compared with the control group, phosphorylated CREB and PGC-1α protein expression were down-regulated significantly by 666-15 incubation (*p* < 0.05) (Figure 7C). Glucagon-induced phosphorylation of CREB and PGC-1α protein expression could be blocked by a pre-treatment with 666-15 (*p* < 0.05). Compared with the control group, 666-15 decreased glucose production in the hepatocytes (*p* > 0.05) but significantly increased the pyruvate concentration in the medium (*p* < 0.05) (Figure 7D). Compared with the glucagon treatment alone, pre-treatment with 666-15 significantly decreased glucose production in the hepatocytes but increased the pyruvate concentration in the medium (*p* < 0.05). No significant difference was observed in glucose concentration in the medium upon different treatments (*p* > 0.05).

### 3.7. SR-18292 Inhibits the Glucagon Pathway in Hepatocytes by Targeting PGC-1α

**Figure 8:**
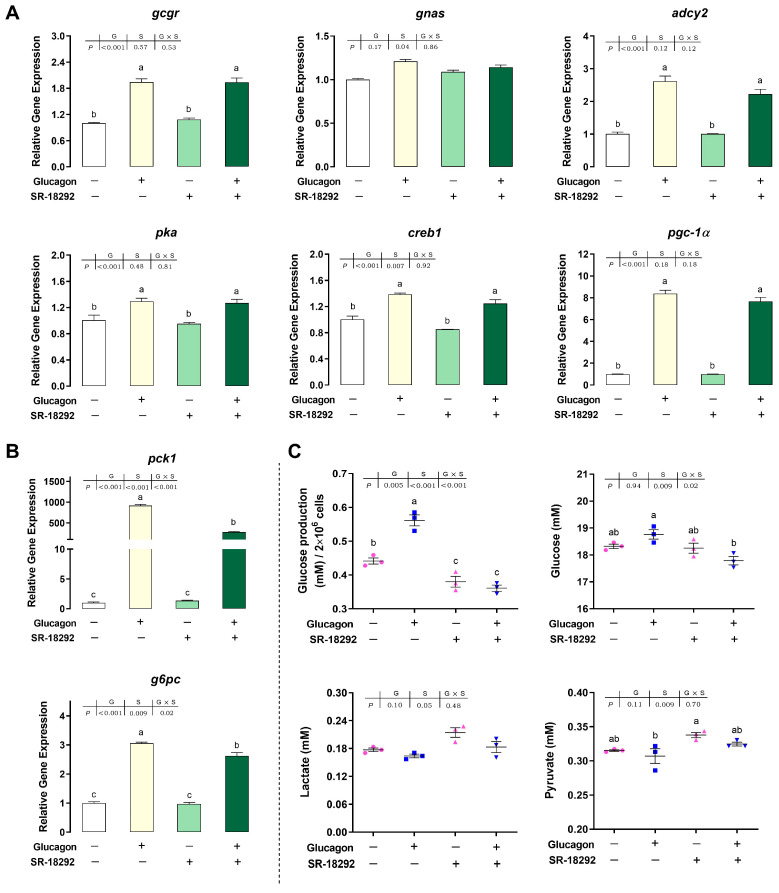
SR-18292 inhibits the glucagon pathway in hepatocytes by targeting PGC-1α. (**A**), qPCR analysis of the mRNA levels of glucagon pathway-related genes (*gcgr*; *gnas*; *adcy2*; *pka*; *creb1*; *pgc-1α*) following glucagon stimulation and treatment with SR-18292 (20 μM) in hepatocytes. (**B**), qPCR analysis of the mRNA levels of gluconeogenic genes (*pck1* and *g6pc*) following glucagon stimulation and treatment with SR-18292 (20 μM) in hepatocytes. (**C**), Glucose production in hepatocytes and glucose, lactate, and pyruvate concentrations in the medium following glucagon stimulation and treatment with SR-18292 (20 μM). All data are expressed as mean ± SE, *n* = 3, two-way ANOVA with Tukey post-hoc test. Values with different letters mean statistical differences (*p* < 0.05). G: glucagon treatments; S: SR-18292 treatments; G×S: interaction between glucagon and SR-18292 treatments.

As shown in Figure 8A,B, compared with the control group, SR-18292 had no effect on the mRNA levels of the measured genes (*p* > 0.05). Following pre-treatment with SR-18292, the stimulatory effects of glucagon on the mRNA levels of *pck1* and *g6pc* were abolished (*p* < 0.05). However, pre-treatment with SR-18292 had no effect on the *gcgr*, *gnas*, *adcy2*, *pka*, *creb1*, and *pgc-1α* mRNA levels compared with glucagon treatment alone (*p* > 0.05). Compared with glucagon treatment, pre-treatment with SR-18292 significantly decreased glucose production in the hepatocytes and the glucose concentration in the medium (*p* < 0.05) (Figure 8C). The lactate and pyruvate concentrations showed upward trends following SR-18292 treatment (*p* > 0.05).

## 4. Discussion

Mammals such as humans, pigs, and rats were found to have only one GCG gene, which encodes several peptide hormones, including glucagon and glucagon-like peptides-1 and -2 (glp1 and glp2) [32]. In the present study, the amino acid sequence of GCG in the Japanese flounder showed more than 50% homology with the corresponding sequences of mammalian GCG. In some teleost, there are two GCG genes with distinct 3′ ends that generate GCG a and b [33]. GCG is a gene that encodes glucagon, glp1, and glp2 and is mainly expressed in the intestine. The GCG b gene encodes glucagon and glp1 and is mainly expressed in the pancreas [34,35]. In the present study, the cloned GCG of the Japanese flounder was shown to share a high sequence similarity with GCG b of other fish species and encodes glucagon and glp1. Glucagon of the Japanese flounder was then chemically synthesized according to the deduced amino acid sequence for incubation with hepatocytes.

In the present study, in vitro incubation with glucagon activated gluconeogenesis and induced glucose production in hepatocytes of the Japanese flounder. In line with the present study, bovine glucagon intraperitoneally injected in blunt snout bream (*Megalobrama amblycephala*) and infused in rainbow trout increased the hepatic glucose production and the mRNA levels of gluconeogenesis-related genes [36,37]. Earlier studies concerning the glucagon signaling pathway in fish found that bovine glucagon bound GCGR (by measuring the radioactivity of hepatocyte-bound ^125^I-labelled glucagon) and increased PKA activity in American eels (*Anguilla rostrata*) and brown bullheads (*Ictalurus nebulosus*) hepatocytes [38,39]. However, the glucagon used in these experiments was not from the fish examined, and the components involved in the signaling pathway were not comprehensively explored. In the present study, more genes downstream of GCGR in the Japanese flounder were investigated; it was found that the mRNA expression of *creb1*, *pgc-1α*, *pck1*, and *g6pc* in the hepatocytes of the Japanese flounder was activated by synthetic glucagon identical to the fish glucagon in a time- and dose-dependent manner.

Adomeglivant is an effective antagonist of GCGR that inhibits the transcription of *gcgr* [40]. In the present study, adomeglivant significantly inhibited the stimulation of the mRNA expression of *gcgr*, *gnas*, *adcy2*, *pka*, *creb1*, *pgc-1α*, *pck1*, *g6pc* and the protein expression of phosphorylated-CREB by glucagon and decreased glucose production in the hepatocytes. Overexpression of GCGR increased the mRNA levels of the above genes, the protein expression of PCK1, and glucose production in the hepatocytes. These results indicated that the CREB1, PGC-1α, PCK1, and G6PC are downstream of GCGR, which is consistent with findings in mammals [3].

In the present study, an inhibitor of PKA (H89) decreased the mRNA expression of *pgc-1α*, *pck1*, *g6pc*, the protein expression of phosphorylated-CREB, and glucose production in the hepatocytes. CREB is a transcription factor residing in the nucleus that is capable of being activated by multiple pathways [41]. In mammals, CREB is activated by glucagon-mediated signaling and induces hepatic gluconeogenesis [42]. However, this characteristic has not been addressed in studies carried out in fish. In grass carp hepatocytes, glucagon treatment could elevate the *creb* mRNA levels and induce CREB phosphorylation [43]. A study in goldfish showed that CREB was activated by glucagon and targeted the leptin gene [44]. To determine the role of CREB in glucagon-mediated gluconeogenesis in the Japanese flounder, 666-15, an antagonist of CREB, was employed to inhibit the transcription of *creb*. We found that 666-15 significantly decreased the stimulation of glucagon on the mRNA expression of *creb1*, *pgc-1α*, and gluconeogenic genes, as well as the protein expression of phosphorylated-CREB and PGC-1α, with glucose production in hepatocytes decreasing accordingly. However, no significant difference in the *gcgr*, *gnas*, *adcy2*, and *pka* mRNA levels was observed following the incubation of the hepatocytes with 666-15. These results demonstrated that CREB is involved in the glucagon-mediated gluconeogenesis pathway. Furthermore, CREB was confirmed to be downstream of PKA and upstream of PGC-1α, G6PC, and PCK1. In mice carrying a targeted disruption of the CREB gene or expressing a dominant negative CREB protein in the liver, the induction of *pgc-1α*, *g6pc*, and *pck1* by glucagon was blocked, and this led to severe hypoglycemia [2]. The results showed that CREB induced the expression of gluconeogenic genes through PGC-1α in response to glucagon stimulation.

The transcriptional co-activator PGC-1α plays a vital role in regulating cellular energy metabolism [45]. Accumulating evidence has indicated that PGC-1α is a key regulator of glucose production and is also an attractive target for anti-diabetic therapy [46,47,48]. However, whether PGC-1α participates in the glucagon-regulated gluconeogenic pathway in fish remains unknown. An antagonist of PGC-1α, SR-18292, which suppresses gluconeogenic gene expression and reduces glucose production in mice hepatocytes [49], was used in the present study. The results indicated that treatment of the hepatocytes with SR-18292 significantly reduced the ability of glucagon to stimulate *pck1* and *g6pc* gene expression and glucose production. This was achieved without disrupting the glucagon-induced increase in the mRNA levels of *gcgr*, *gnas*, *adcy2*, *pka*, and *creb1*. These results indicated that PGC-1α is downstream of the glucagon-mediated CREB signaling pathway and mediates the activation of gluconeogenesis in the Japanese flounder.

## 5. Conclusions

**Figure 9:**
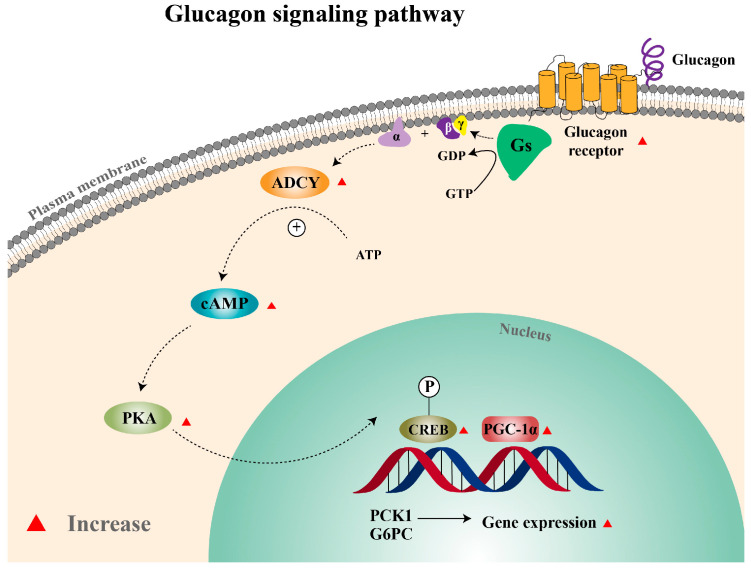
Glucagon signaling pathway in the Japanese flounder. Gs, guanine nucleotide-binding protein Gs; GDP, guanine dinucleotide phosphate; GTP, guanine trinucleotide phosphate; ADCY, adenylate cyclase; ATP, adenosine triphosphate; cAMP, cyclic adenosine monophosphate; PKA, protein kinase A; CREB, cAMP response element-binding protein; PGC-1α, peroxisome proliferator-activated receptor-γ coactivator 1-α; PCK1, phosphoenolpyruvate carboxykinase 1; G6PC, glucose-6-phosphatase.

In summary, the present study confirmed that glucagon promoted gluconeogenesis through the GCGR/PKA/CREB/PGC-1α pathway in the Japanese flounder (Figure 9). These results illustrate the presence of similar pathways regulating gluconeogenesis by glucagon in the Japanese flounder and mammals. They provide a basis for determining whether glucagon-regulated gluconeogenesis is one of the reasons for glucose intolerance in carnivorous fish and further finding targets to improve this disorder.

## Figures and Tables

**Table 1:** Sequences of the PCR primers used for cloning, plasmid construction, and qPCR analysis.

Primer	Sequences (5′-3′)	Accession Number
Primers for partial cDNA		
^a^ GCG-F	YGGACTCCTGCTCYTCAT	MW727222
GCG-R	AACTCCTTGGCKGCCTGG	
Primers for RACE PCR		
5′-GCG-R1 GGTTCGGTCATCATGGAGT	GGTTCGGTCATCATGGAGT	MW727222
5′-GCG-R2	GATGAGAAGCAGGAGTCCG	
3′-GCG-F1	CGAGCACAAGACTTTGTCCA	
3′-GCG-F2	ATGCAGACGGCACCTACAC	
UPM (long)	ACTCACTATAGGGCAAGCAGTGGTATCAACGCAGAGT	
UPM (short)	CTAATACGACTACTATAGGGC	
NUP	AAGCAGTGGTATCAACGCAGAGT	
Primers for GCGR cds		
^b^ GCGR-F	GAGCAGCGGTGAAGAGCAG	KY742714
GCGR-R	ATGTCAGGGCCAGACGAGA	
Primers with homology arms		
GCGR-h-F	5′-cttggtaccgagctcggatccATGT CACGGCTGTTCCTCCTT-3′	KY742714
GCGR-h-R	5′-atggtggcgaccggtggatccCAGGTG GCTCTCTGCGTTCT-3′	
Primers for qPCR		
^a^ *gcg*-q-F	CGGACTCCTGCTTCTCAT	MW727222
*gcg*-q-R	TTTGCTGCCTTGTCTTGC	
^b^ *gcgr*-q-F	AGGACGTTTGACAATTATGC	KY742714
*gcgr*-q-R	TCTTCGTAGCCACCCACT	
^c^ *gnas*-q-F	TCCGGCAGACTGACTACAC	KY742715
*gnas*-q-R	GACCTCCAACATCAAACATA	
^d^ *adcy2*-q-F	AGAGTGATGAAAGCCGAAAT	KY742716
*adcy2*-q-R	TGGTCAAACTTGCCGAAC	
^e^ *pka*-q-F	GCATTGGACCACTTTGAG	KY742718
*pka*-q-R	GACAGCTTGCAGGATACG	
^f^ *creb1*-q-F	CGCAGGAAGAAGAAGGAGT	MT215340
*creb1*-q-R	GGTCTTTAAGGGCTTTGAGT	
^g^ *pgc-1α*-q-F	GGATTGCCTTCGTTTGAGG	MT215340
*pgc-1α*-q-R	GCTGGCTGCATGGTTCTG	
^h^ *g6pc*-q-F	TACCCCGTGACCTGTGAGAC	KY742717
*g6pc*-q-R	TTGGTGGATTTCTTGCTTCC	
^i^ *pck1*-q-F	ACCAGGGACCAGAAGGACA	MN173838
*pck1*-q-R	TGGCGACCACGTAGGGAGA	
*β-actin*-q-F	GGAAATCGTGCGTGACATTAAG	HQ386788
*β-actin*-q-R	CCTCTGGACAACGGAACCTCT	

^a ^GCG/*gcg*, proglucagon. ^b^ GCGR/*gcgr*, glucagon receptor. ^c^ *gnas*, guanine nucleotide-binding protein Gs subunit alpha. ^d^ *adcy2*, adenylate cyclase 2. ^e^ *pka*, protein kinase A. ^f^ *creb1*, cyclic AMP-responsive element-binding protein 1. ^g^ *pgc-1α*, peroxisome proliferator-activated receptor-γ coactivator 1-α. ^h^ *g6pc*, glucose-6-phosphatase. ^i^ *pck1*, phosphoenolpyruvate carboxykinase 1.

## Data Availability

The data that support the findings of this study are available from the corresponding author upon reasonable request.

## References

[1] Park M.K., Takei Y., Ando H., Tsutsui K. (2016). Glucagon. Handbook of Hormones.

[2] Herzig S., Long F.X., Jhala U.S., Hedrick S., Quinn R., Bauer A., Rudolph D., Schutz G., Yoon C., Puigserver P. (2001). CREB regulates hepatic gluconeogenesis through the coactivator PGC-1. Nature.

[3] Jiang G., Zhang B.B. (2003). Glucagon and regulation of glucose metabolism. Am. J. Physiol. Endocrinol. Metab..

[4] Forbes J.M., Cooper M.E. (2013). Mechanisms of diabetic complications. Physiol. Rev..

[5] Richardson L.C., Pollack L.A. (2005). Therapy insight: Influence of type 2 diabetes on the development, treatment and outcomes of cancer. Nat. Clin. Pract. Oncol..

[6] Lund A., Bagger J.I., Christensen M., Knop F.K., Vilsbøll T. (2014). Glucagon and type 2 diabetes: The return of the alpha cell. Curr. Diabetes Rep..

[7] D’alessio D. (2011). The role of dysregulated glucagon secretion in type 2 diabetes. Diabetes Obes. Metab..

[8] Godoy-Matos A.F. (2014). The role of glucagon on type 2 diabetes at a glance. Diabetol. Metab. Syndr..

[9] Yan H., Gu W., Yang J., Bi V., Shen Y., Lee E., Winters K.A., Komorowski R., Zhang C., Patel J.J. (2009). Fully human monoclonal antibodies antagonizing the glucagon receptor improve glucose homeostasis in mice and monkeys. J. Pharmacol. Exp. Ther..

[10] Hare K.J., Vilsboll T., Asmar M., Deacon C.F., Knop F.K., Holst J.J. (2010). The glucagonostatic and insulinotropic effects of glucagon-like peptide 1 contribute equally to its glucose-lowering action. Diabetes.

[11] Liang Y., Osborne M.C., Monia B.P., Bhanot S., Gaarde W.A., Reed C., She P.X., Jetton T.L., Demarest K.T. (2004). Reduction in glucagon receptor expression by an antisense oligonucleotide ameliorates diabetic syndrome in db/db mice. Diabetes.

[12] Yang H., Yang L. (2016). Targeting cAMP/PKA signaling pathway for glycemic control and type 2 diabetes therapy. J. Mol. Endocrinol..

[13] Furuichi M., Yone Y. (1981). Change of blood sugar and plasma insulin levels of fishes in glucose tolerance test. Nippon. Suisan Gakkaishi.

[14] Malik M.A., Komarewar S., Dar S.A. (2020). Why fish is a natural diabetic animal?. World J. Aquac Res. Dev..

[15] Kamalam B.S., Panserat S. (2016). Carbohydrates in fish nutrition. Int. Aquafeed..

[16] Su J.Z., Gong Y.L., Mei L.Y., Xi L.W., Chi S.Y., Yang Y.X., Jin J.Y., Liu H.K., Zhu X.M., Xie S.Q. (2020). The characteristics of glucose homoeostasis in grass carp and Chinese longsnout catfish after oral starch administration: A comparative study between herbivorous and carnivorous species of fish. Br. J. Nutr..

[17] Viegas I., Mendes V.M., Leston S., Jarak I., Carvalho R.A., Pardal M.A., Manadas B., Jones J.G. (2011). Analysis of glucose metabolism in farmed European sea bass (*Dicentrarchus labrax* L.) using deuterated water. Comp. Biochem. Physiol. A-Mol. Integr. Physiol..

[18] Higuera M., Cardenas P. (1986). Hormonal effects on gluconeogenesis from (U-14C) glutamate in rainbow trout (*Salmo gairdneri*). Comp. Biochem. Physiol. B-Biochem. Mol. Biol..

[19] Ottolenghi C., Puviani A.C., Baruffaldi A., Gavioli M.E., Brighenti L. (1988). Glucagon control of glycogenolysis in catfish tissues. Comp. Biochem. Physiol. B-Biochem. Mol. Biol..

[20] Deng K., Pan M.Z., Liu J.H., Yang M.X., Gu Z.X., Zhang Y., Liu G.X., Liu D., Zhang W.B., Mai K.S. (2018). Chronic stress of high dietary carbohydrate level causes inflammation and influences glucose transport through SOCS3 in Japanese flounder *Paralichthys olivaceus*. Sci. Rep..

[21] Liu D., Deng K.Y., Sampath W.W.H.A., Gu Z.X., Pan M.Z., Zhang Y., Zhang W.B., Mai K.S. (2019). Responses of glucosensing system to glucose in Japanese flounder *Paralichthys olivaceus* fed diets with different carbohydrate content. Comp. Biochem. Physiol. B-Biochem. Mol. Biol..

[22] Liu J.H., Deng K.Y., Pan M.Z., Liu G.X., Wu J., Yang M.X., Huang D., Zhang W.B., Mai K.S. (2020). Dietary carbohydrates influence muscle texture of olive flounder *Paralichthys olivaceus* through impacting mitochondria function and metabolism of glycogen and protein. Sci. Rep..

[23] Yang M.X., Deng K.Y., Pan M.Z., Gu Z.X., Liu D., Zhang Y., Zhang W.B., Mai K.S. (2019). Glucose and lipid metabolic adaptations during postprandial starvation of Japanese flounder *Paralichthys olivaceus* previously fed different levels of dietary carbohydrates. Aquaculture.

[24] Yang M.X., Deng K.Y., Pan M.Z., Zhang Y., Sampath W.W.H.A., Zhang W.B., Mai K.S. (2019). Molecular adaptations of glucose and lipid metabolism to different levels of dietary carbohydrates in juvenile Japanese flounder *Paralichthys olivaceus*. Aquac. Nutr..

[25] Pan M.Z., Zhang Y., Deng K.Y., Liu G.X., Gu Z.X., Liu J.H., Luo K., Zhang W.B., Mai K.S. (2019). Forkhead box O1 in turbot *Scophthalmus maximus*: Molecular characterization, gene structure, tissue distribution and the role in glucose metabolism. Gene.

[26] Gu Z., Pan M., Liu J., Yang M., Zhang W., Mai K. (2022). Molecular cloning of AKT1 and AKT2 and their divergent responses to insulin and glucose at transcriptional level in the liver of Japanese flounder *Paralichthys olivaceus*. Aquac. Rep..

[27] Peng Y.H., Wang P., He X.Q., Hong M.Z., Liu F. (2022). Micro ribonucleic acid-363 regulates the phosphatidylinositol 3-kinase/threonine protein kinase axis by targeting NOTCH1 and forkhead box C2, leading to hepatic glucose and lipids metabolism disorder in type 2 diabetes mellitus. J. Diabetes Investig..

[28] MacQueen J., Plaut D. (1979). Colorimetric microdetermination of plasma lactate. Am. J. Med. Technol..

[29] Xie H.L., Zhu S., Zhang J., Wen J., Yuan H.J., Pan L.Z., Luo M.J., Tan J.H. (2018). Glucose metabolism during in vitro maturation of mouse oocytes: An study using RNA interference. J. Cell. Physiol..

[30] Sun C., Wang M.H., Liu X.Y., Luo L., Li K.X., Zhang S.Q., Wang Y.J., Yang Y.M., Ding F., Gu X.S. (2014). PCAF improves glucose homeostasis by suppressing the gluconeogenic activity of PGC-1α. Cell Rep..

[31] Liu J.H., Pan M.Z., Huang D., Guo Y.L., Yang M.X., Zhang W.B., Mai K.S. (2020). Myostatin-1 inhibits cell proliferation by inhibiting the mTOR signal pathway and MRFs, and activating the ubiquitin-proteasomal system in skeletal muscle cells of Japanese flounder *Paralichthys olivaceus*. Cells.

[32] Irwin D.M. (2002). Ancient duplications of the human proglucagon gene. Genomics.

[33] Cardoso J., Félix R., Costa C., Palma P., Canário A., Power D.M. (2017). Evolution of the glucagon-like system across fish. Gen. Comp. Endocrinol..

[34] Plisetskaya E.M., Mommsen T.P. (1996). Glucagon and glucagon-like peptides in fishes. Int. Rev. Cytol..

[35] Zhou L., Irwin D.M. (2004). Fish proglucagon genes have differing coding potential. Comp. Biochem. Physiol. B-Biochem. Mol. Biol..

[36] Forbes J.L.I., Kostyniuk D.J., Mennigen J.A., Weber J.M. (2019). Glucagon regulation of carbohydrate metabolism in rainbow trout: In vivo glucose fluxes and gene expression. J. Exp. Biol..

[37] Shi H.J., Liu W.B., Xu C., Zhang L., Liu J., Zhang D.D., Zhang L., Li X.F. (2020). Transcriptional regulation of the AMP-activated protein kinase and glycolipid metabolism-related genes by insulin and glucagon in blunt snout bream (*Megalobrama amblycephala*): A comparative study. Aquaculture.

[38] Moon T.W., Gambarotta A., Capuzzo A., Fabbri E. (1997). Glucagon and glucagon-like peptide signaling pathways in the liver of two fish species, the American eel and the black bullhead. J. Exp. Zool..

[39] Navarro I., Moon T.W. (1994). Glucagon binding to hepatocytes isolated from two teleost fishes, the American eel and the brown bullhead. J. Endocrinol..

[40] Kazda C.M., Ying D., Kelly R.P., Garhyan P., Hardy T.A. (2016). Evaluation of efficacy and safety of the glucagon receptor antagonist LY2409021 in patients with type 2 diabetes: 12- and 24-week phase 2 studies. Diabetes Care.

[41] Gonzalez G.A., Montminy M.R. (1989). Cyclic AMP stimulates somatostatin gene transcription by phosphorylation of CREB at serine 133. Cell.

[42] Oh K.J., Han H.S., Kim M.J., Koo S.H. (2013). CREB and FoxO1: Two transcription factors for the regulation of hepatic gluconeogenesis. Bmb Rep..

[43] Bai J., Jiang X., He M., Chan B., Wong A. (2019). Novel mechanisms for IGF-I regulation by glucagon in carp hepatocytes: Up-regulation of HNF1α and CREB expression via signaling crosstalk for IGF-I gene transcription. Front. Endocrinol..

[44] Yan A.F., Chen T., Shuang C., Tang D.S., Hu C.Q. (2016). Signal transduction mechanism for glucagon-induced leptin gene expression in goldfish liver. Int. J. Biol. Sci..

[45] Corona J.C., Duchen M.R. (2015). PPARγ and PGC-1α as therapeutic targets in Parkinson’s. Neurochem. Res..

[46] Rhee J., Inoue Y., Yoon J.C., Puigserver P., Fan M., Gonzalez F.J., Spiegelman B.M. (2003). Regulation of hepatic fasting response by PPARγ coactivator-1α (PGC-1): Requirement for hepatocyte nuclear factor 4α in gluconeogenesis. Proc. Natl. Acad. Sci. USA.

[47] Wu H., Deng X., Shi Y., Ye S., Duan H. (2016). PGC-1α, glucose metabolism and type 2 diabetes mellitus. J. Endocrinol..

[48] Yoon J.C., Pulgserver P., Chen G., Donovan J., Wu Z.D., Rhee J., Adelmant G., Stafford J., Kahn C.R., Granner D.K. (2001). Control of hepatic gluconeogenesis through the transcriptional coactivator PGC-1. Nature.

[49] Sharabi K., Hua L., Tavares C., Dominy J.E., Puigserver P. (2017). Selective chemical inhibition of PGC-1α gluconeogenic activity ameliorates type 2 diabetes. Cell.

